# Optimal neoadjuvant regimens for locally advanced gastric and gastroesophageal junction cancer: a systematic review and bayesian network meta-analysis

**DOI:** 10.1186/s12957-025-04151-z

**Published:** 2025-12-24

**Authors:** Wei-Qiang Fan, Ya-Ting Wu, Hang Li, Hui Xu, Yun-Yun Zhu, Yan-Ran Cheng, Yong-Hai Peng

**Affiliations:** 1 Department of Oncology, The CangShan District of the 900th Hospital, Fuzhou, Fujian 350000 China; 2 Department of Radiation Oncology, 900th Hospital of PLA Joint Logistics Support Force, Fuzhou, Fujian 350000 China

**Keywords:** Stomach neoplasms, Esophagogastric junction, Neoadjuvant therapy, Network Meta-Analysis, Immunotherapy, Resection rate

## Abstract

**Background:**

Large randomized controlled trials (RCTs) have reported data on neoadjuvant therapies for locally advanced gastric cancer (GC) and gastroesophageal junction cancer (GEJC). However, no clear recommendation is provided regarding the optimal choice of neoadjuvant regimen. This network meta-analysis (NMA) endeavors to identify the best neoadjuvant therapy for the locally advanced GC and GEJC.

**Methods:**

PubMed, Embase, Cochrane Library, and Web of Science databases were searched systematically until September 6, 2024 and update until November 14, 2025. This study encompassed RCTs investigating neoadjuvant treatment for locally advanced GC or GEJC that reported outcomes on overall survival (OS), pathological complete response (pCR), as well as R0 resection rates. Study quality was rated utilizing the Cochrane Risk of Bias Tool 2.0. Bayesian network meta-analysis (BNMA) was conducted through Stata and RStudio for efficacy comparison and ranking of all regimens.

**Results:**

26 RCTs comprising 7,324 patients with GC or GEJC were encompassed. Through the surface under the cumulative ranking curve (SUCRA), DOX ranked highest for OS. FLOT combined with Durvalumab (FLOT_Durvalumab) demonstrated a superior pCR rate, while DOS combined with apatinib (DOS_Apatinib) ranked highest for R0 resection rate. DOX significantly prolonged OS compared with CS (HR = 0.54, 95% CrI: 0.29–0.99), surgery (HR = 0.49, 95% CrI: 0.29–0.84), and XELOX (HR = 0.59, 95% CrI: 0.35–0.99).

**Conclusions:**

DOX appears to be the most effective neoadjuvant treatment for ameliorating OS in patients with GC or GEJC. FLOT_Durvalumab was associated with the highest pCR rate, while DOS_Apatinib appears most effective in achieving R0 resection.

**Supplementary Information:**

The online version contains supplementary material available at 10.1186/s12957-025-04151-z.

## Introduction

Gastric cancer (GC) and gastroesophageal junction cancer (GEJC) rank among the deadliest cancers globally. Epidemiological data indicate that GC constitutes nearly 5% of all malignancies by incidence and death, with a particularly heavy disease burden in Asia [1]. Due to the intricate nature of its early symptoms, an estimated 50%−60% of the patients with GC/GEJC are identified only after progressing to an unresectable or metastatic stage, thereby complicating treatment, eventually resulting in a generally poor prognosis [2]. Surgical resection remains the primary approach for treating locally advanced GC/GEJC. Nevertheless, surgery alone often proves insufficient due to the significant likelihood of both local recurrence and metastatic spread, with five-year survival rates falling below 30%. Multidisciplinary treatment approaches are essential to ameliorate clinical outcomes [3].

The MAGIC trial proved the importance of neoadjuvant therapy in the management of GC/GEJC. Compared to surgery, perioperative chemotherapy involving epirubicin, cisplatin, and fluorouracil (ECF) notably ameliorated progression-free survival (PFS) and overall survival (OS) [4]. High-quality clinical trials further proved the clinical significance of neoadjuvant chemotherapy and chemoradiotherapy alongside continuous refinements to therapeutic regimens over time [3, 5–7]. Immunotherapy has opened new avenues for neoadjuvant strategies in locally advanced GC/GEJC. For patients with microsatellite instability-high or mismatch repair-deficient (MSI-H/dMMR) tumors, neoadjuvant immunotherapy has gained increasing clinical endorsement [8–11]. Among unselected patient populations, neoadjuvant regimens incorporating immune checkpoint inhibitors (ICIs) significantly improve R0 resection rates and increase pathological complete response (pCR) rates. For instance, the KEYNOTE-585 trial demonstrated that the locally advanced gastroesophageal adenocarcinoma patients, perioperative or adjuvant chemotherapy plus pembrolizumab, followed by adjuvant pembrolizumab, markedly improved the pCR compared to chemotherapy plus placebo (13.0% vs. 2.4%) [12]. In the DANTE/FLOT8 trial, toripalimab plus neoadjuvant chemotherapy outperformed chemotherapy alone, yielding better pCR (22.2% vs. 7.4%) and R0 resection (94.4% vs. 92.6%) rates [13]. Similarly, the DANTE/IKF-s633 study found that atezolizumab plus FLOT as perioperative therapy in the resectable esophagogastric adenocarcinoma (EGA) population notably enhanced both pCR (24% vs. 15%) and R0 resection rates (96% vs. 95%) [14].

Despite the abundance of studies evaluating the efficacy of neoadjuvant therapies, direct, high-quality comparisons among different treatment modalities remain limited. Existing meta-analyses of neoadjuvant immunotherapy for GC/GEJC are predominantly conventional in design. Wang et al. reported that neoadjuvant immunotherapy yielded promising short-term efficacy and favorable safety profiles in resectable GC/GEJC [15]. Yu et al. further proved that integrating PD-1 inhibitors into neoadjuvant chemotherapy increased the probability of curative surgical resection and ameliorated prognosis in the locally advanced gastric cancer (LAGC) population [15, 16]. Unlike prior pairwise meta-analyses, this study is the first to employ a Bayesian network meta-analysis to generate a comprehensive ranking of neoadjuvant regimens. Bayesian network meta-analysis (BNMA) is effective in the simultaneous comparison of multiple therapeutic strategies, thereby providing more comprehensive evidence to support clinical decision-making. Accordingly, the present study seeks to compare neoadjuvant chemotherapy, chemoradiotherapy, and immunotherapy in treating locally advanced GC/GEJC through a systematic review and BNMA. Through this comprehensive synthesis, this study endeavors to provide more precise clinical recommendations and guide future research directions.

## Methods

### Study design and registration

Our systematic review followed the Preferred Reporting Items for Systematic Reviews and Meta-Analyses (PRISMA) [17] and was prospectively registered in the International Prospective Register of Systematic Reviews (PROSPERO, CRD42024590225, https://www.crd.york.ac.uk/PROSPERO/view/CRD42024590225). The work has been reported in line with AMSTAR 2 (Assessing the methodological quality of systematic reviews) Guidelines [18].

### Literature retrieval

Two independent reviewers retrieved PubMed, Embase, Cochrane Library, and Web of Science up to September 6, 2024 and update until November 14, 2025. Both Medical Subject Headings (MeSH) and free-text keywords were utilized in the search, encompassing all known variants of terms such as “Esophagogastric Junction,” “gastroesophageal junction cancer,” “stomach cancer,” “Stomach Neoplasms,” and “neoadjuvant therapy.” The strategy is detailed in Supplementary Material 1.

### Eligibility criteria

As per the PICOS framework, our inclusion criteria were: (1) Population: Patients aged ≥ 18 pathologically diagnosed with GC or GEJC; (2) Intervention: The intervention or control group received neoadjuvant therapy; (3) Outcomes: Inclusion of RCTs that demonstrate R0 resection rate or number, pCR, or OS; (4) Study Design: Randomized controlled trials (RCTs); (5) Language: English.

Our exclusion criteria were: (1) Population: Patients with metastatic or unresectable disease; (2) Study Type: Cohort or descriptive studies, reviews, case reports, opinion pieces, conference abstracts, or exploratory cohort; (3) Data Availability: Insufficient data for synthesis; (4) Accessibility: Studies without full texts.

### Data extraction

Two reviewers screened the studies independently as per the predefined eligibility criteria. All identified records were first imported into EndNote X9 for deduplication. Subsequently, titles and abstracts were screened, and eligible full texts were reviewed for final study inclusion. Dissents were addressed through discussion with a third reviewer.

Extracted data were the first author, publication year, geographic region, demographic characteristics, interventions, registration number, and extractable outcome indicators. Outcomes, including R0 resection rate, pCR, and OS, were extracted.

### Risk of bias assessment

The risk of bias in eligible RCTs was rated through the Cochrane Risk of Bias Tool 2.0 [19]. The assessment involved bias from the randomization procedure, deviations from prescribed interventions, missing outcomes, outcome measurement, and selective result reports encompassing protocol-linked biases. Each was rated as “low risk of bias,” “some concerns,” or “high risk of bias.” Studies with any high-risk domains were considered high risk overall, whereas only those with all low-risk domains were deemed low risk.

### Data analysis

For dichotomous outcomes (R0 resection and pCR), relative risks (RRs) with 95% credible intervals (CrIs) were computed. Regarding OS, hazard ratios (HRs) with 95% CrIs were extracted. When survival data were unavailable but survival curves were provided, HRs and 95% CrIs were extracted through Engauge Digitizer according to the method described by Tierney et al. [20]. Network plots were generated utilizing Stata 15.1. Funnel plots presented possible publication bias. BNMA was conducted using the “Gemtc” package 1.0–2 in R 4.3.2. Four Markov chains were run for each outcome with 50,000 iterations per chain, discarding the first 20,000 iterations as burn-in. Heterogeneity was quantified through the I² statistic, where a value below 50% denoted low heterogeneity, whereas 50% or more signified substantial heterogeneity. The random-effects model should be adopted as the primary analytical approach. A fixed-effects model is recommended only under specific circumstances: when the network is sparse and the random-effects model fails to converge, and when heterogeneity is considered low (I² < 50%) [21, 22]. The deviance information criterion (DIC) was utilized to evaluate model fit and assess consistency across direct and indirect evidence by comparing consistency and inconsistency models. A DIC difference of < 5 denoted a good model fit, in which case the consistency model was adopted [23]. The convergence of Markov chains was examined through trace plots and the Gelman-Rubin-Brooks method, where a potential scale reduction factor (PSRF) approaching 1 suggested satisfactory convergence. Further validation involved surface under the cumulative ranking curve (SUCRA), league tables, and consistency checks. SUCRA values indicate the probability that a given treatment ranks highest in efficacy: 1 denotes absolute efficacy and 0 suggests no efficacy. Higher SUCRA values indicate superior intervention rankings [24]. In this network meta-analysis, all cisplatin-based fluoropyrimidine doublet chemotherapy regimens (including cisplatin plus capecitabine [XP] and cisplatin plus fluorouracil [FP]) were considered a single intervention node, based on established evidence of their equivalent efficacy [25]. To ensure network connectivity, the chemotherapeutic backbones from the KEYNOTE-585 main cohort (which used both XP and FP) were pooled and defined as fluorouracil-cisplatin (FC). The separate FLOT cohort from KEYNOTE-585 was excluded from this analysis, as it constituted an exploratory safety cohort with a distinct chemotherapeutic backbone and was not designed for primary efficacy assessment.

## Results

### Literature selection process

**Fig. 1:**
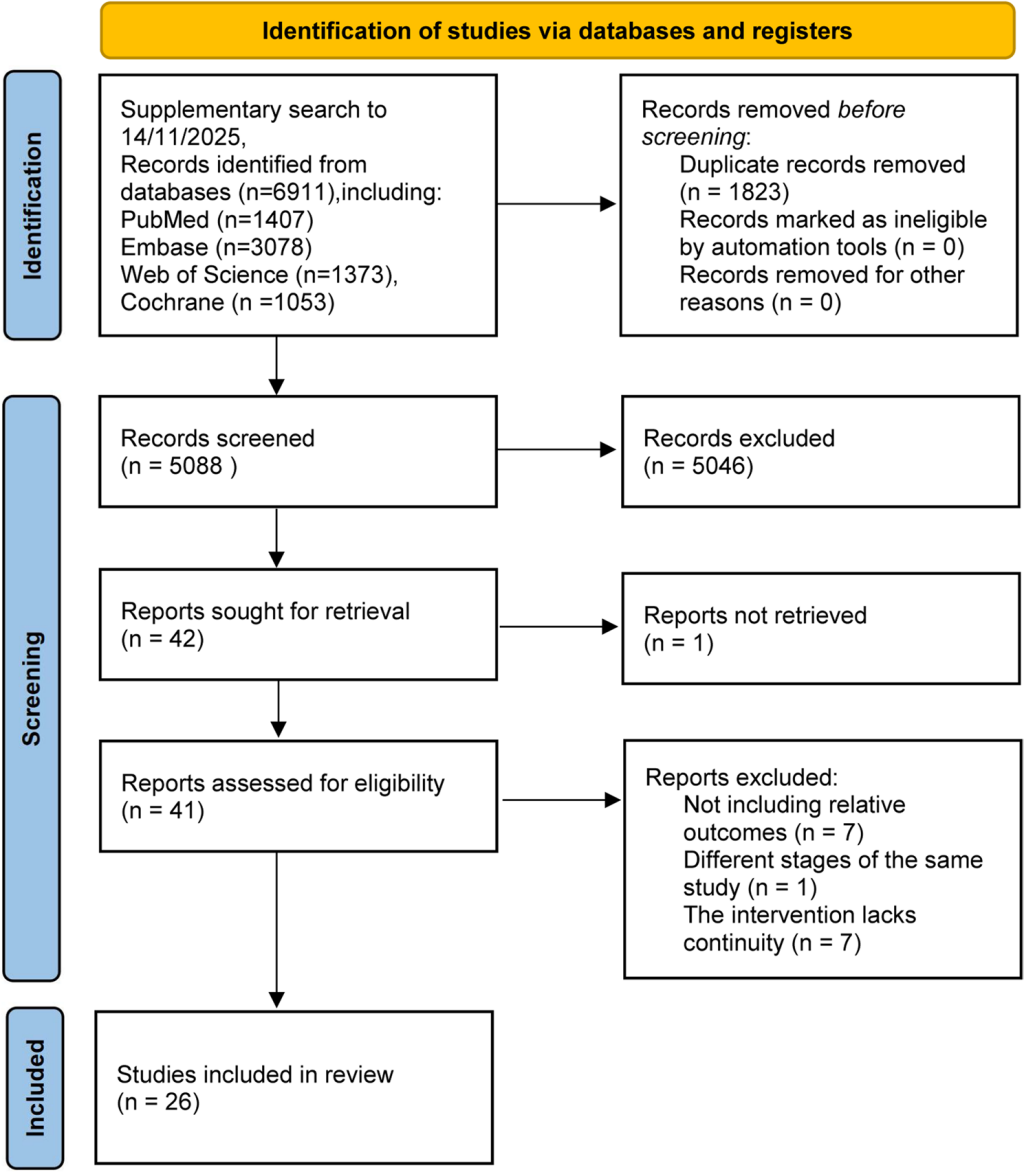
Article screening flowchart

The selection process is presented in Fig. 1. Initially, a total of 6,911 potentially relevant articles were identified through database searches up to November 14, 2025. After the exclusion of 1,823 duplicates, the titles and abstracts of the rest were checked as per the foregoing eligibility criteria. 5,046 studies were subsequently excluded. Primary exclusion criteria included: meta-analyses, reviews, study protocols, case reports, non-English publications, studies not meeting the PICOS criteria, animal studies, letters, and commentaries. And the full texts of the remaining 42 studies were checked. Of these, 16 were excluded due to the absence of relevant outcome measures, being different phases of the same study, inconsistency in the intervention regimens, or unavailability of full texts (Fig. 1). Ultimately, 26 RCTs were incorporated [4, 5, 11–14, 26–46].

### Study characteristics

A total of 22 neoadjuvant regimens were evaluated in our NMA, as detailed in Table S1. The characteristics and specific information of eligible studies are summarized in Table 1. Across 26 RCTs, a total of 7,234 GC or GEJC patients were included, with a varying number of subjects from 15 up to 474 per study and mean ages between 56 and 67.05 years. Specifically, 12 encompassed GC patients [26, 29, 31–33, 35–37, 39–42], 13 enrolled patients with GC/GEJC [4, 5, 12–14, 27, 28, 34, 38, 43–46], and one included only GEJC patients [37]. Regarding neoadjuvant strategies, four studies incorporated radiotherapy [30, 37, 39, 41], and five studies combined chemotherapy with immunotherapy [12–14, 45, 46], while the remaining involved chemotherapy alone. In terms of outcomes, 15 studies reported R0 resection rates [5, 13, 14, 28, 32, 34, 35, 38–43, 45, 46], 10 reported pCR [12–14, 28, 34, 41–43, 45, 46], and 18 provided OS data [4, 5, 12, 26, 27, 29–33, 35–37, 39, 41–44]. KEYNOTE-585 was excluded from the pCR analysis because its intervention regimen constituted a disconnected node in the network meta-analysis. Ultimately, the pooled analysis for pCR included a total of 9 studies.

**Table 1:** Characteristics of the study

First Author	Year	Phase	Disease	Sample Size (*n*)				Media Age(years)				Neoadjuvant Protocol				Outcomes	Trial Number
				Arm1	Arm2	Arm3	Arm4	Arm1	Arm2	Arm3	Arm4	Arm1	Arm2	Arm3	Arm4		
Cunningham	2006	Ⅲ	G/GEJ	250	250	-	-	62	62	-	-	ECF	None	-	-	OS	ISRCTN93793971
Imano	2010	-	G	16	15	16	16	61.5	60.5	58.4	59.5	F	C	FC	None	OS	-
Schuhmache	2010	Ⅲ	G/GEJ	72	72	-	-	56	58	-	-	FC	None	-	-	OS	NCT00004099
Al-Batran	2016	Ⅱ-Ⅲ	G/GEJ	137	128	-	-	62	64	-	-	ECF	FLOT	-	-	R0/pCR	NCT01216644
Fazio	2015	Ⅲ	G	34	35	-	-	57	59	-	-	DCF	None	-	-	OS	-
Stahl	2017	Ⅲ	GEJ	59	60	-	-	56	60.6	-	-	FC	FC_RT	-	-	OS	-
Zhao	2017	Ⅱ-Ⅲ	G	50	52	-	-	59	58.5	-	-	SOX	None	-	-	OS	-
Xue	2018	Ⅱ-Ⅲ	G	25	25	-	-	None	None	-	-	SOX	XELOX	-	-	R0/OS	ISRCTN12206108
Al-Batran	2019	Ⅱ-Ⅲ	G/GEJ	360	356	-	-	62	62	-	-	FLOT	ECF	-	-	R0/OS	NCT01216644
Hayashi	2020	Ⅱ	G	32	31	-	-	66.5	61	-	-	DCS	CS	-	-	OS	UMIN000006387
Sah	2020	Ⅱ	G/GEJ	40	34	-	-	67	61	-	-	FLOT	SOX	-	-	R0/pCR	NCT03636893
Zhao	2020	Ⅲ	G	223	236	290	-	None	None	None	-	SOX	XELOX	None	-	R0/OS	NCT01516944
Iwasaki	2020	Ⅲ	G	151	149	-	-	64	62	-	-	CS	None	-	-	OS	UMINC000000279
Yuan Tian	2021	Ⅱ-Ⅲ	GEJ	76	73	-	-	64	65	-	-	XELOX_RT	None	-	-	OS	NCT01962246
Tian	2021	Ⅱ	G/GEJ	100	100	100	-	None	None	None	-	XELOX	DOX	None	-	R0	NCT02555358
Wang	2021	-	G	30	30	-	-	None	None	-	-	XELOX_RT	XELOX	-	-	R0/OS	-
Zhou	2021	-	G	40	40	-	-	67.05	65.14	-	-	DOS_Apatinib	DOS	-	-	R0	-
Wang	2022	Ⅱ	G	37	38	-	-	58	57	-	-	SOX_RT	SOX	-	-	R0/pCR/OS	NCT02301481
Janjigian	2023	Ⅲ	G/GEJ	474	474	-	-	None	None	-	-	FLOT_Durvalumab	FLOT	-	-	R0/pCR	NCT04592913
Li	2023	Ⅲ	G/GEJ	180	180	-	-	63	63	-	-	SOX_Apatinib_Camrelizumab	SOX	-	-	R0/pCR	NCT04208347
Tian	2023	Ⅲ	G	100	100	100	-	None	None	None	-	DOX	XELOX	None	-	R0/pCR/OS	NCT02555358
Jiang	2024	Ⅱ	G/GEJ	71	76	-	-	61	58	-	-	DOS	SOX	-	-	R0/pCR/OS	NCT02725424
Kang	2024	Ⅲ	G/GEJ	246	238	-	-	58	58	-	-	DOS	None	-	-	OS	NCT01515748
Lorenzen	2024	Ⅱ	G/GEJ	146	149	-	-	61	62	-	-	FLOT_Atezolizumab	FLOT	-	-	R0/pCR	NCT03421288
Yuan	2024	Ⅱ	G/GEJ	54	54	-	-	58	62	-	-	SOX_Toripalimab	SOX	-	-	R0/pCR	NCT04250948
Shitara	2025	Ⅲ	G/GEJ	402	402	-	-	64	63	-	-	FC_Pembrolizumab	FC	-	-	PCR/OS	NCT03221426

*C* Cisplatin, *CS* Cisplatin, S-1, *DCF* Docetaxel, Cisplatin, Fluorouracil, *DCS* Docetaxel, Cisplatin, S-1, *DOS* Docetaxel, Oxaliplatin, S-1, *DOS*_*Apatinib* Docetaxel, Oxaliplatin, S-1, Apatinib, *DOX* Docetaxel, Oxaliplatin, Capecitabine, *ECF* Epirubicin, Cisplatin, Fluorouracil, *F* Fluorouracil, *FC* Fluorouracil, Cisplatin, *FC_RT* Fluorouracil, Cisplatin, Radiotherapy, *FLOT* Fluorouracil, Leucovorin, Oxaliplatin, Docetaxel, *FLOT_Atezolizumab* Fluorouracil, Leucovorin, Oxaliplatin, Docetaxel, Atezolizumab, *FLOT_Durvalumab * Fluorouracil, Leucovorin, Oxaliplatin, Docetaxel, Durvalumab, *FLOT_Pembrolizumab * Fluorouracil, Leucovorin, Oxaliplatin, Docetaxel, Pembrolizumab, *SAP* S-1, Nab-paclitaxel, *SAP_Apatinib_Camrelizumab* S-1, Nab-paclitaxel, Apatinib, Camrelizumab, *SOX* S-1,Oxaliplatin, *SOX_Apatinib_Camrelizumab* S-1, Oxaliplatin, Apatinib, Camrelizumab, *SOX_RT* S-1, Oxaliplatin, Radiotherapy, *SOX_Toripalimab* S-1, Oxaliplatin, Toripalimab, *XELOX* Capecitabine, Oxaliplatin, *XELOX_RT* Capecitabine, Oxaliplatin, Radiotherapy, *FC* Fluorouracil, Cisplatin

### Quality assessment

Figure S3 presents the risk-of-bias assessment results. Among 26 eligible RCTs, 61.5% exhibited low risk of bias overall, while 38.5% were considered to have potential risk. Possible sources of bias included the randomization process (*n* = 4; 19.2%), deviations from prescribed interventions (*n* = 9; 34.2%), missing outcomes (*n* = 1; 3.8%), and outcome measurement (*n* = 1; 3.8%). All other domains were rated as low risk.

### Outcome measures

#### OS

**Fig. 2:**
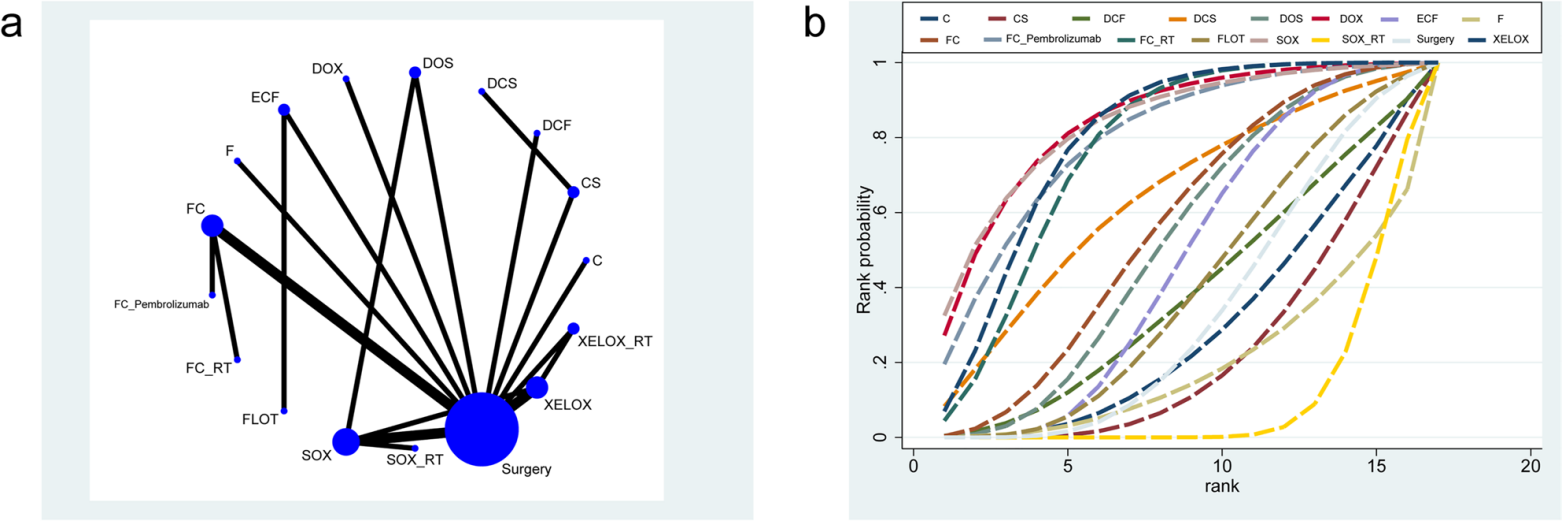
Mesh and SUCA diagrams for OS. **a** OS Mesh Diagram, (**b**) OS (SUCRA)

A total of 18 RCTs involving 17 neoadjuvant regimens assessed their effects on OS (network diagram shown in Fig. 2a). The corresponding league table is provided in Table 2. DOX (SUCRA 84%, top-rank) significantly prolonged OS compared to CS (HR = 0.54, 95%CrI: 0.29–0.99), surgery(HR = 0.49, 95%CrI: 0.29–0.84), and XELOX(HR = 0.59, 95%CrI: 0.35–0.99), respectively(Fig. 2b).

**Table 2:** OS league table

**C**	1.05 (0.63, 1.74)	0.93 (0.51, 1.7)	0.72 (0.36, 1.48)	0.83 (0.5, 1.37)	0.57 (0.29, 1.11)	0.86 (0.54, 1.36)	1.12 (0.68, 1.83)	0.94 (0.64, 1.37)	0.81 (0.53, 1.24)	0.61 (0.34, 1.09)	0.66 (0.4, 1.09)	0.91 (0.54, 1.54)	0.56 (0.26, 1.17)	1.15 (0.76, 1.72)	0.96 (0.58, 1.56)	0.63 (0.37, 1.06)
0.95 (0.57, 1.58)	**CS**	0.88 (0.52, 1.52)	0.69 (0.42, 1.14)	0.79 (0.52, 1.2)	**0.54 (0.29**,** 0.99)**	0.82 (0.56, 1.19)	1.06 (0.59, 1.91)	0.89 (0.57, 1.4)	0.77 (0.47, 1.26)	0.58 (0.31, 1.09)	**0.63 (0.41**,** 0.96)**	0.87 (0.55, 1.35)	0.53 (0.27, 1.05)	1.09 (0.81, 1.47)	0.91 (0.61, 1.37)	**0.6 (0.38**,** 0.94)**
1.08 (0.59, 1.98)	1.13 (0.66, 1.94)	**DCF**	0.78 (0.37, 1.63)	0.89 (0.52, 1.53)	0.61 (0.3, 1.23)	0.93 (0.56, 1.52)	1.2 (0.61, 2.36)	1.01 (0.58, 1.77)	0.87 (0.48, 1.57)	0.66 (0.32, 1.34)	0.71 (0.42, 1.22)	0.98 (0.56, 1.71)	0.6 (0.28, 1.29)	1.23 (0.79, 1.93)	1.03 (0.61, 1.74)	0.68 (0.39, 1.19)
1.38 (0.68, 2.81)	1.45 (0.88, 2.4)	1.28 (0.61, 2.69)	**DCS**	1.14 (0.59, 2.2)	0.78 (0.35, 1.73)	1.19 (0.64, 2.22)	1.54 (0.71, 3.34)	1.3 (0.66, 2.54)	1.12 (0.55, 2.24)	0.84 (0.38, 1.88)	0.91 (0.48, 1.76)	1.26 (0.64, 2.46)	0.77 (0.33, 1.8)	1.58 (0.88, 2.84)	1.32 (0.69, 2.52)	0.87 (0.45, 1.71)
1.21 (0.73, 2)	1.27 (0.83, 1.94)	1.12 (0.65, 1.93)	0.87 (0.45, 1.69)	**DOS**	0.68 (0.37, 1.25)	1.04 (0.72, 1.51)	1.35 (0.75, 2.43)	1.13 (0.73, 1.77)	0.98 (0.6, 1.59)	0.74 (0.4, 1.38)	0.8 (0.52, 1.22)	1.1 (0.78, 1.56)	0.67 (0.36, 1.27)	**1.38 (1.03**,** 1.87)**	1.16 (0.79, 1.69)	0.76 (0.49, 1.18)
1.77 (0.9, 3.48)	**1.86 (1.01**,** 3.44)**	1.65 (0.82, 3.31)	1.28 (0.58, 2.83)	1.47 (0.8, 2.68)	**DOX**	1.52 (0.85, 2.73)	1.98 (0.94, 4.16)	1.66 (0.89, 3.14)	1.43 (0.74, 2.79)	1.08 (0.5, 2.34)	1.17 (0.63, 2.17)	1.61 (0.88, 2.94)	0.98 (0.44, 2.18)	**2.03 (1.19**,** 3.48)**	**1.69 (1.01**,** 2.86)**	1.12 (0.62, 2.02)
1.16 (0.74, 1.84)	1.22 (0.84, 1.77)	1.08 (0.66, 1.78)	0.84 (0.45, 1.57)	0.96 (0.66, 1.39)	0.66 (0.37, 1.18)	**ECF**	1.3 (0.75, 2.25)	1.09 (0.74, 1.63)	0.94 (0.6, 1.46)	0.71 (0.39, 1.29)	**0.77 (0.63**,** 0.94)**	1.06 (0.71, 1.58)	0.65 (0.33, 1.25)	**1.33 (1.07**,** 1.66)**	1.11 (0.78, 1.58)	0.73 (0.49, 1.09)
0.9 (0.55, 1.47)	0.94 (0.52, 1.7)	0.83 (0.42, 1.64)	0.65 (0.3, 1.41)	0.74 (0.41, 1.34)	0.51 (0.24, 1.06)	0.77 (0.44, 1.34)	**F**	0.84 (0.52, 1.37)	0.72 (0.43, 1.22)	0.55 (0.28, 1.05)	0.59 (0.33, 1.07)	0.81 (0.45, 1.49)	0.5 (0.22, 1.11)	1.03 (0.62, 1.71)	0.86 (0.48, 1.53)	0.56 (0.31, 1.04)
1.06 (0.73, 1.55)	1.12 (0.71, 1.75)	0.99 (0.57, 1.73)	0.77 (0.39, 1.51)	0.88 (0.56, 1.38)	0.6 (0.32, 1.13)	0.92 (0.62, 1.36)	1.19 (0.73, 1.93)	**FC**	0.86 (0.7, 1.05)	0.65 (0.42, 1.01)	0.7 (0.45, 1.1)	0.97 (0.61, 1.55)	0.59 (0.29, 1.19)	1.22 (0.88, 1.7)	1.02 (0.66, 1.57)	0.67 (0.42, 1.07)
1.24 (0.81, 1.9)	1.3 (0.8, 2.12)	1.15 (0.64, 2.08)	0.9 (0.45, 1.81)	1.02 (0.63, 1.67)	0.7 (0.36, 1.36)	1.06 (0.68, 1.66)	1.38 (0.82, 2.34)	1.16 (0.95, 1.42)	**FC_Pembrolizumab**	0.76 (0.47, 1.23)	0.82 (0.5, 1.33)	1.13 (0.68, 1.88)	0.69 (0.33, 1.43)	1.42 (0.96, 2.09)	1.18 (0.73, 1.91)	0.78 (0.47, 1.3)
1.64 (0.92, 2.91)	1.72 (0.92, 3.21)	1.52 (0.75, 3.1)	1.19 (0.53, 2.65)	1.36 (0.72, 2.53)	0.92 (0.43, 2)	1.41 (0.78, 2.54)	1.83 (0.95, 3.52)	1.54 (0.99, 2.38)	1.32 (0.81, 2.14)	**FC_RT**	1.09 (0.58, 2.02)	1.49 (0.78, 2.83)	0.91 (0.4, 2.08)	**1.88 (1.08**,** 3.25)**	1.57 (0.84, 2.9)	1.03 (0.54, 1.96)
1.51 (0.92, 2.49)	**1.59 (1.04**,** 2.42)**	1.4 (0.82, 2.4)	1.09 (0.57, 2.11)	1.25 (0.82, 1.91)	0.85 (0.46, 1.58)	**1.3 (1.06**,** 1.59)**	1.69 (0.94, 3.03)	1.42 (0.91, 2.22)	1.22 (0.75, 1.99)	0.92 (0.49, 1.73)	**FLOT**	1.38 (0.88, 2.15)	0.84 (0.42, 1.67)	**1.73 (1.29**,** 2.33)**	1.45 (0.96, 2.17)	0.95 (0.61, 1.49)
1.1 (0.65, 1.85)	1.15 (0.74, 1.8)	1.02 (0.58, 1.78)	0.8 (0.41, 1.55)	0.91 (0.64, 1.29)	0.62 (0.34, 1.13)	0.94 (0.63, 1.4)	1.23 (0.67, 2.24)	1.03 (0.65, 1.65)	0.89 (0.53, 1.47)	0.67 (0.35, 1.28)	0.73 (0.47, 1.13)	**SOX**	0.61 (0.36, 1.04)	1.26 (0.9, 1.75)	1.05 (0.74, 1.49)	0.69 (0.45, 1.06)
1.8 (0.86, 3.78)	1.89 (0.95, 3.76)	1.67 (0.78, 3.59)	1.3 (0.56, 3.05)	1.49 (0.79, 2.8)	1.02 (0.46, 2.25)	1.55 (0.8, 2.99)	2.01 (0.9, 4.5)	1.69 (0.84, 3.41)	1.45 (0.7, 3.03)	1.1 (0.48, 2.53)	1.19 (0.6, 2.37)	1.64 (0.97, 2.77)	**SOX_RT**	**2.06 (1.11**,** 3.84)**	1.72 (0.91, 3.23)	1.14 (0.57, 2.23)
0.87 (0.58, 1.31)	0.92 (0.68, 1.24)	0.81 (0.52, 1.27)	0.63 (0.35, 1.13)	**0.72 (0.53**,** 0.97)**	**0.49 (0.29**,** 0.84)**	**0.75 (0.6**,** 0.93)**	0.97 (0.59, 1.62)	0.82 (0.59, 1.14)	0.7 (0.48, 1.04)	**0.53 (0.31**,** 0.93)**	**0.58 (0.43**,** 0.78)**	0.79 (0.57, 1.11)	**0.48 (0.26**,** 0.9)**	**Surgery**	0.83 (0.63, 1.1)	**0.55 (0.39**,** 0.77)**
1.05 (0.64, 1.71)	1.1 (0.73, 1.65)	0.97 (0.57, 1.64)	0.76 (0.4, 1.44)	0.87 (0.59, 1.26)	**0.59 (0.35**,** 0.99)**	0.9 (0.63, 1.28)	1.17 (0.65, 2.09)	0.98 (0.64, 1.52)	0.84 (0.52, 1.36)	0.64 (0.35, 1.18)	0.69 (0.46, 1.04)	0.95 (0.67, 1.35)	0.58 (0.31, 1.09)	1.2 (0.91, 1.58)	**XELOX**	0.66 (0.48, 0.91)
1.59 (0.94, 2.68)	**1.66 (1.06**,** 2.61)**	1.47 (0.84, 2.58)	1.15 (0.59, 2.24)	1.31 (0.85, 2.02)	0.9 (0.5, 1.62)	1.36 (0.91, 2.03)	1.77 (0.96, 3.26)	1.49 (0.93, 2.39)	1.28 (0.77, 2.14)	0.97 (0.51, 1.84)	1.05 (0.67, 1.64)	1.44 (0.94, 2.22)	0.88 (0.45, 1.74)	**1.82 (1.3**,** 2.54)**	**1.52 (1.1**,** 2.1)**	**XELOX_RT**

#### pCR

**Fig. 3:**
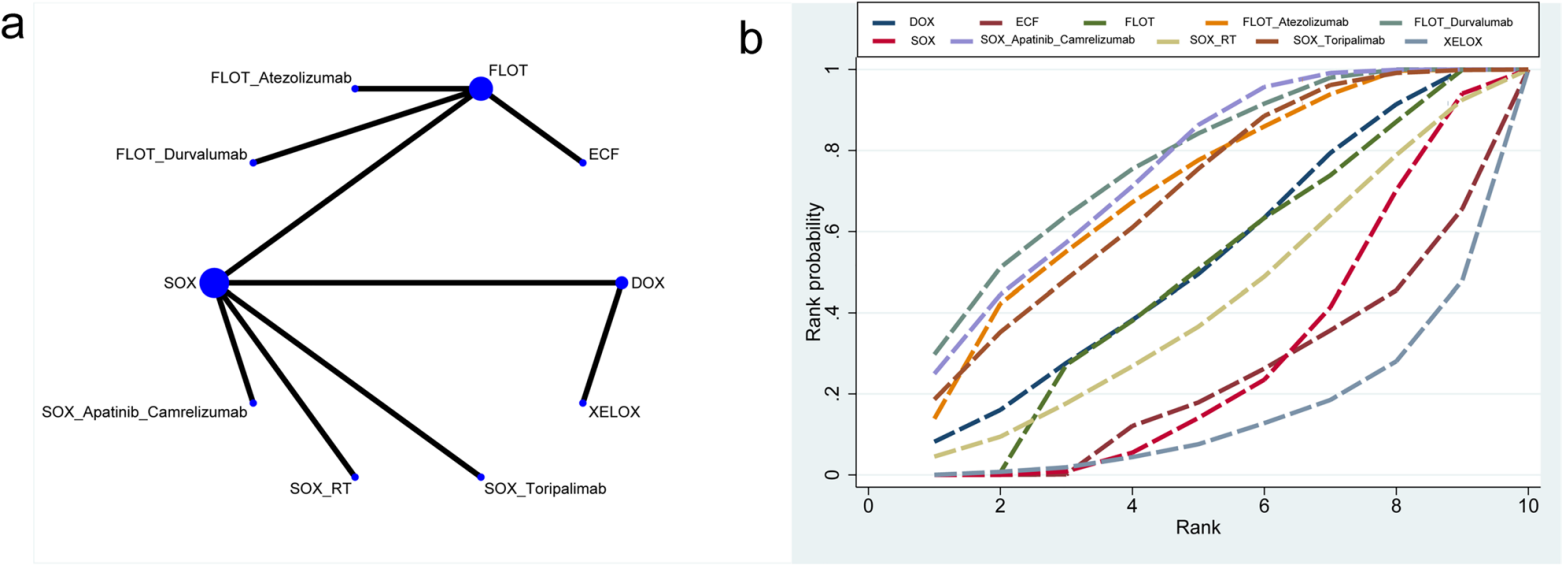
Mesh and SUCA diagrams for pCR. **a** pCR mesh, (**b**) pCR(SUCRA)

A total of 9 RCTs involving 10 neoadjuvant regimens evaluated their effects on pCR (network diagram shown in Fig. 3a). The corresponding league table is presented in Table 3. FLOT_Durvalumab(SUCRA 77%, top-rank) significantly improved pCR rates compared to ECF(RR = 5.15, 95%CrI: 2.31–12.68), FLOT(RR = 1.87, 95%CrI: 1.44–2.45), respectively(Fig. 3b).

**Table 3:** pCR league table

**DOX**	0.39 (0.02, 16.18)	1.09 (0.06, 42.06)	1.79 (0.09, 71.37)	2.04 (0.1, 79.07)	0.53 (0.1, 2.19)	2.06 (0.34, 10.6)	0.76 (0.09, 6.58)	1.69 (0.25, 10.88)	0.25 (0.07, 0.69)
2.54 (0.06, 56.02)	**ECF**	**2.75 (1.3**,** 6.52)**	**4.51 (1.84**,** 12.13)**	**5.15 (2.31**,** 12.68)**	1.35 (0.04, 19.07)	5.24 (0.15, 82.68)	1.92 (0.05, 42.37)	4.3 (0.12, 78.33)	0.62 (0.01, 16.54)
0.92 (0.02, 18.03)	**0.36 (0.15**,** 0.77)**	**FLOT**	**1.63 (1.01**,** 2.7)**	**1.87 (1.44**,** 2.45)**	0.49 (0.02, 6.03)	1.89 (0.06, 26.2)	0.69 (0.02, 13.69)	1.56 (0.05, 24.84)	0.22 (0.01, 5.34)
0.56 (0.01, 11.41)	**0.22 (0.08**,** 0.54)**	**0.61 (0.37**,** 0.99)**	**FLOT_Atezolizumab**	1.15 (0.65, 1.98)	0.3 (0.01, 3.84)	1.15 (0.03, 16.65)	0.42 (0.01, 8.72)	0.95 (0.03, 15.89)	0.14 (0.01, 3.38)
0.49 (0.01, 9.73)	**0.19 (0.08**,** 0.43)**	**0.54 (0.41**,** 0.7)**	0.87 (0.51, 1.54)	**FLOT_Durvalumab**	0.26 (0.01, 3.24)	1.01 (0.03, 14.14)	0.37 (0.01, 7.37)	0.83 (0.02, 13.49)	0.12 (0.01, 2.89)
1.88 (0.46, 9.66)	0.74 (0.05, 23.63)	2.04 (0.17, 61.15)	3.35 (0.26, 103.2)	3.82 (0.31, 115.03)	**SOX**	**3.83 (1.87**,** 8.89)**	1.43 (0.33, 7.27)	**3.13 (1.15**,** 10.84)**	0.47 (0.07, 3.21)
0.49 (0.09, 2.9)	0.19 (0.01, 6.58)	0.53 (0.04, 16.97)	0.87 (0.06, 28.62)	0.99 (0.07, 32.36)	**0.26 (0.11**,** 0.53)**	**SOX_Apatinib_Camrelizumab**	0.37 (0.07, 2.19)	0.82 (0.22, 3.41)	**0.12 (0.02**,** 0.96)**
1.32 (0.15, 11.75)	0.52 (0.02, 21.51)	1.44 (0.07, 56.11)	2.36 (0.11, 94.52)	2.69 (0.14, 105.5)	0.7 (0.14, 3.07)	2.7 (0.46, 14.66)	**SOX_RT**	2.22 (0.32, 14.93)	0.33 (0.03, 3.71)
0.59 (0.09, 4.07)	0.23 (0.01, 8.49)	0.64 (0.04, 22.07)	1.05 (0.06, 37.4)	1.2 (0.07, 41.63)	**0.32 (0.09**,** 0.87)**	1.22 (0.29, 4.52)	0.45 (0.07, 3.11)	**SOX_Toripalimab**	0.15 (0.02, 1.32)
**3.94 (1.46**,** 13.94)**	1.61 (0.06, 77.45)	4.47 (0.19, 202.23)	7.31 (0.3, 339.47)	8.35 (0.35, 382.56)	2.13 (0.31, 13.94)	**8.23 (1.04**,** 63.48)**	3.06 (0.27, 36)	6.79 (0.76, 62.01)	**XELOX**

#### R0

**Fig. 4:**
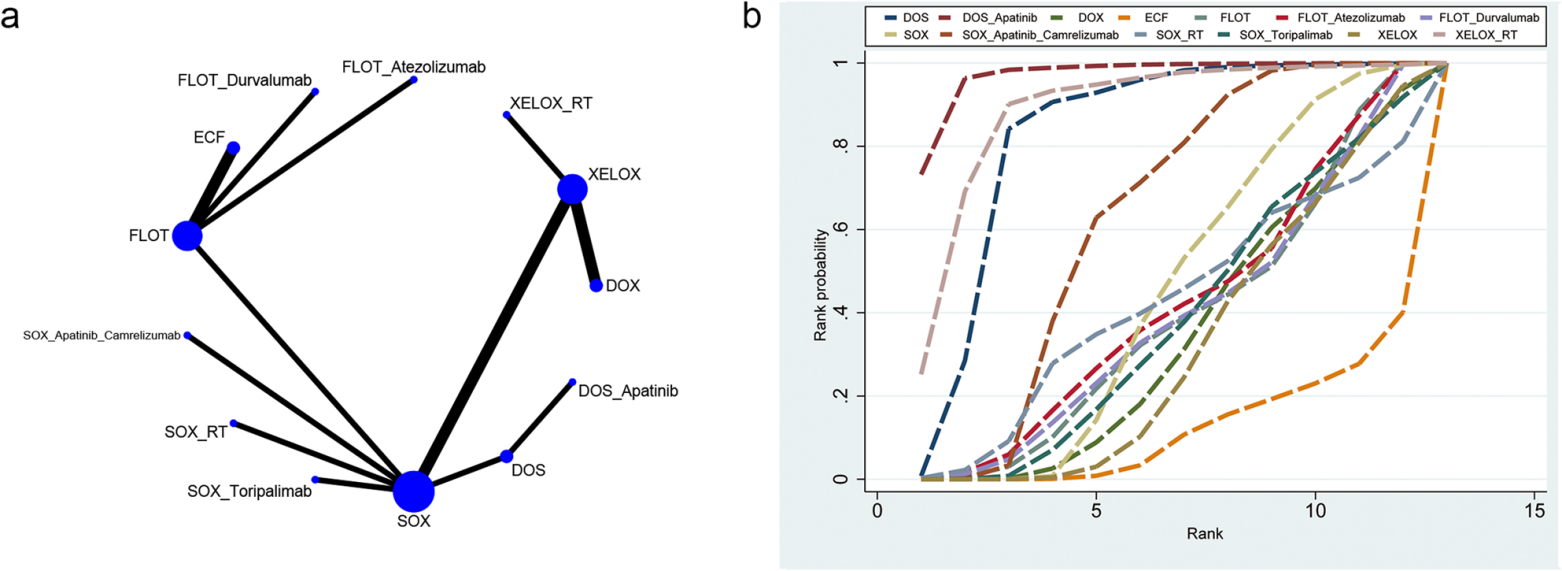
Mesh and SUCA diagrams for R0. **a** R0 mesh, (**b**) R0(SUCRA)

A total of 15 RCTs involving 13 neoadjuvant regimens were evaluated for their effects on R0 resection rates (network diagram shown in Fig. 4a). The league table is presented in Table 4. The DOS_Apatinib(SUCRA 97%, top-rank) regimen resulted in a significantly higher R0 resection rate compared to DOX(RR = 1.77, 95%CrI: 1.2–2.69), ECF(RR = 1.99, 95%CrI: 1.2–3.34), FLOT(RR = 1.8, 95%CrI: 1.09–3.01), FLOT_Atezolizumab(RR = 1.78, 95%CrI: 1.08–2.99), FLOT_Durvalumab(RR = 1.8, 95%CrI: 1.08–3.01), SOX(RR = 1.72, 95%CrI: 1.18–2.59), SOX_Apatinib_Camrelizumab(RR = 1.64, 95%CrI: 1.12–2.47), SOX_RT(RR = 1.74, 95%CrI: 1.08–2.86), SOX_Toripalimab(RR = 1.76, 95%CrI: 1.19–2.68), and XELOX(RR = 1.78, 95%CrI: 1.21–2.69) 1.78 (1.21–2.69), respectively(Fig. 4b).

**Table 4:** R0 league table

**DOS**	1.35 (0.99, 1.9)	0.76 (0.6, 0.96)	0.68 (0.46, 1.01)	0.75 (0.51, 1.11)	0.76 (0.51, 1.13)	0.75 (0.51, 1.12)	0.79 (0.62, 0.97)	0.82 (0.65, 1.02)	0.78 (0.54, 1.11)	0.77 (0.6, 0.98)	0.76 (0.6, 0.95)	1.13 (0.74, 1.79)
0.74 (0.53, 1.01)	**DOS_Apatinib**	0.56 (0.37, 0.83)	0.5 (0.3, 0.84)	0.56 (0.33, 0.92)	0.56 (0.33, 0.93)	0.56 (0.33, 0.92)	0.58 (0.39, 0.85)	0.61 (0.4, 0.89)	0.57 (0.35, 0.92)	0.57 (0.37, 0.84)	0.56 (0.37, 0.82)	0.83 (0.49, 1.45)
**1.31 (1.04**,** 1.67)**	**1.77 (1.2**,** 2.69)**	**DOX**	0.89 (0.64, 1.26)	0.99 (0.71, 1.39)	0.99 (0.71, 1.41)	0.99 (0.71, 1.4)	1.03 (0.95, 1.13)	1.08 (0.98, 1.19)	1.02 (0.75, 1.37)	1.01 (0.87, 1.16)	1 (0.95, 1.05)	**1.48 (1.04**,** 2.22)**
1.47 (0.99, 2.18)	**1.99 (1.2**,** 3.34)**	1.12 (0.79, 1.57)	**ECF**	**1.11 (1.04**,** 1.18)**	**1.11 (1.03**,** 1.21)**	**1.11 (1.02**,** 1.2)**	1.15 (0.83, 1.6)	1.21 (0.86, 1.68)	1.14 (0.73, 1.75)	1.13 (0.79, 1.59)	1.12 (0.79, 1.55)	**1.66 (1.02**,** 2.77)**
1.33 (0.9, 1.96)	**1.8 (1.09**,** 3.01)**	1.01 (0.72, 1.41)	0.9 (0.85, 0.96)	**FLOT**	1.01 (0.95, 1.06)	1 (0.95, 1.06)	1.04 (0.75, 1.43)	1.09 (0.78, 1.51)	1.03 (0.67, 1.58)	1.02 (0.72, 1.43)	1.01 (0.72, 1.4)	1.5 (0.92, 2.5)
1.32 (0.89, 1.95)	**1.78 (1.08**,** 2.99)**	1.01 (0.71, 1.4)	0.9 (0.83, 0.97)	0.99 (0.94, 1.05)	**FLOT_Atezolizumab**	0.99 (0.92, 1.07)	1.03 (0.74, 1.43)	1.08 (0.77, 1.5)	1.02 (0.66, 1.57)	1.01 (0.71, 1.43)	1 (0.71, 1.39)	1.49 (0.91, 2.49)
1.33 (0.89, 1.97)	**1.8 (1.08**,** 3.01)**	1.01 (0.72, 1.41)	0.9 (0.83, 0.98)	1 (0.95, 1.06)	1.01 (0.93, 1.09)	**FLOT_Durvalumab**	1.04 (0.75, 1.44)	1.09 (0.78, 1.51)	1.03 (0.66, 1.58)	1.02 (0.72, 1.44)	1.01 (0.72, 1.4)	1.5 (0.92, 2.51)
**1.27 (1.03**,** 1.6)**	**1.72 (1.18**,** 2.59)**	0.97 (0.89, 1.06)	0.87 (0.63, 1.21)	0.96 (0.7, 1.34)	0.97 (0.7, 1.35)	0.96 (0.7, 1.34)	**SOX**	**1.05 (1.003**,** 1.1)**	0.99 (0.74, 1.32)	0.98 (0.87, 1.1)	0.97 (0.9, 1.03)	**1.43 (1.01**,** 2.17)**
1.22 (0.98, 1.53)	**1.64 (1.12**,** 2.47)**	0.93 (0.84, 1.02)	0.83 (0.6, 1.16)	0.92 (0.66, 1.28)	0.92 (0.67, 1.29)	0.92 (0.66, 1.28)	0.96 (0.91, 0.997)	**SOX_Apatinib_Camrelizumab**	0.95 (0.7, 1.26)	0.94 (0.82, 1.06)	0.92 (0.85, 1)	1.37 (0.96, 2.08)
1.29 (0.9, 1.86)	**1.74 (1.08**,** 2.86)**	0.98 (0.73, 1.33)	0.88 (0.57, 1.36)	0.97 (0.63, 1.5)	0.98 (0.64, 1.52)	0.97 (0.63, 1.51)	1.01 (0.76, 1.35)	1.06 (0.79, 1.42)	**SOX_RT**	0.99 (0.73, 1.35)	0.98 (0.73, 1.32)	1.45 (0.92, 2.39)
**1.3 (1.02**,** 1.68)**	**1.76 (1.19**,** 2.68)**	0.99 (0.86, 1.15)	0.88 (0.63, 1.26)	0.98 (0.7, 1.39)	0.99 (0.7, 1.41)	0.98 (0.7, 1.4)	1.02 (0.91, 1.15)	1.07 (0.95, 1.21)	1.01 (0.74, 1.37)	**SOX_Toripalimab**	0.99 (0.86, 1.13)	**1.46 (1.01**,** 2.25)**
**1.32 (1.05**,** 1.67)**	**1.78 (1.21**,** 2.69)**	1 (0.95, 1.06)	0.9 (0.64, 1.26)	0.99 (0.72, 1.39)	1 (0.72, 1.41)	0.99 (0.71, 1.4)	1.03 (0.97, 1.11)	1.08 (0.999, 1.18)	1.02 (0.76, 1.37)	1.01 (0.89, 1.16)	**XELOX**	**1.48 (1.05**,** 2.23)**
0.89 (0.56, 1.35)	1.2 (0.69, 2.06)	0.68 (0.45, 0.96)	0.6 (0.36, 0.98)	0.67 (0.4, 1.08)	0.67 (0.4, 1.09)	0.67 (0.4, 1.09)	0.7 (0.46, 0.99)	0.73 (0.48, 1.04)	0.69 (0.42, 1.08)	0.68 (0.44, 0.99)	0.67 (0.45, 0.95)	**XELOX_RT**

#### Serious adverse event (SAE)

Five studies reported Grade ≥ 3 treatment-linked adverse events and the types of common SAEs (Table S2). The incidence of SAEs across these regimens ranged from 24.2% to 35.2%. The SOX_Toripalimab (35.2%), SAP_Apatinib_Camrelizumab (33.3%), and XELOX (32.6%) regimens demonstrated the highest SAE rates, respectively. The most frequently observed SAEs were thrombocytopenia (18.5%) in the SOX_Toripalimab regimen, leukopenia (13.7%) in the SAP_Apatinib_Camrelizumab regimen, and neutropenia (30.4%) in the XELOX regimen.

### Assessment of consistency and publication bias

The consistency and inconsistency models were compared through DIC. All models exhibited a DIC variation of less than 5, indicating good overall consistency. In the analysis of OS, closed loops formed among neoadjuvant regimens permitted local consistency assessment through node-splitting. The P-values for all comparisons were > 0.05, suggesting the absence of local inconsistency (Figure S1).

Concerning publication bias, a comparison of adjusted funnel plots (Figures S2a-2c) revealed no evidence of publication bias.

## Discussion

Our analysis demonstrates that DOX confers the greatest improvement in OS, that FLOT_Durvalumab achieves the highest pCR rate, and that DOS_Apatinib attains the most favorable R0 resection rate. It is important to emphasize that SUCRA values indicate the probability of a treatment being the most effective among the compared interventions, rather than serving as definitive evidence of statistical superiority. These rankings should be interpreted with caution, especially when SUCRA values are closely clustered, as such differences may not correspond to clinically meaningful distinctions.

In the phase III NCT02555358 trial, the neoadjuvant DOX regimen demonstrated a statistically significant improvement in OS comparison to XELOX, with a HR of 0.64 (95% CI: 0.42–0.97). This outcome indicates a clinically meaningful survival benefit in patients with locally advanced gastric adenocarcinoma [42]. The DOX regimen, comprising docetaxel, oxaliplatin, and capecitabine, establishes a synergistic multi‑targeted therapeutic framework by integrating non‑cell cycle-specific and cell cycle-specific agents. Docetaxel stabilizes microtubules and disrupts mitotic spindle formation. Oxaliplatin induces interstrand DNA cross-links, and capecitabine inhibits thymidylate synthase to impair DNA synthesis. The foregoing complementary mechanisms enhance the clearance of circulating tumor cells and micrometastases, which may underlie the observed OS advantage. This finding is further supported by a meta-analysis by Chi et al., who identified DOX/DOS as the optimal neoadjuvant regimen for improving OS in LAGC, thereby corroborating our conclusion [47].

Our NMA results on pCR further prove the efficacy of neoadjuvant immunotherapy, with FLOT_Durvalumab emerging as the most effective regimen. Notably, all four immunotherapy-containing strategies among the 10 analyzed ranked highly by SUCRA. A recent meta-analysis by Yu et al. focusing on immunotherapy-containing neoadjuvant regimens also demonstrated that such combinations significantly enhance pCR [16], which is consistent with our findings. Immunotherapy-based combination strategies have likewise been associated with improved pCR rates in other gastrointestinal malignancies. In the SPRING-01 trial, which enrolled patients with locally advanced rectal cancer, the addition of immunotherapy to neoadjuvant chemoradiotherapy led to a markedly higher pCR rate compared with the control group (59.2% vs. 32.7%) [48]. Similarly, a meta-analysis by Liu et al. evaluating neoadjuvant therapy for resectable esophageal cancer demonstrated that combining immunotherapy with chemoradiotherapy significantly increased the pCR rate [49]. Moreover, we identified FLOT_Durvalumab as the most effective regimen among the immunotherapy-based options. This can be explained by the high efficacy of FLOT alone among non-immunotherapy regimens [50], along with the Durvalumab. Durvalumab augments the FLOT regimen by counteracting tumor-induced immunosuppression through PD-L1 blockade, thereby restoring T cell-mediated tumor elimination. with chemotherapy-induced immunogenic cell death and remodeling of the tumor microenvironment, this immunologic reactivation enhances antitumor activity and contributes to the observed improvement in pCR rates [51].

Regarding R0 resection, our NMA results identified DOS_Apatinib as the most effective regimen. Zhou et al. reported a 77.5% R0 resection rate with neoadjuvant DOS_Apatinib for locally advanced GC [40]. DOS_Apatinib comprises docetaxel, oxaliplatin, and S-1 combined with apatinib, a tyrosine kinase inhibitor. In Asia, DOS is commonly used as neoadjuvant therapy. In the DOCET_R_05153 study, the R0 resection rate with neoadjuvant DOS among GC/GEJC patients was 95% compared to 84% in the surgery-taking cohort. Apatinib, a VEGFR-2 TKI, exhibits clinical efficacy in third-line treatment of advanced GC/GEJC by significantly ameliorating PFS and OS, and is recommended in clinical practice [52–54]. Moreover, the single-arm 2017YF004-03 study reported a 75% R0 resection rate with SOX plus apatinib as neoadjuvant treatment in locally advanced GC [55]. Collectively, these findings prove the superior performance of DOS_Apatinib in improving R0 resection rates. It is important to note that the key clinical evidence supporting the combination S1 regimen is primarily derived from studies conducted in Asian populations [44, 56]. Extrapolation of these findings to broader, non-Asian populations requires further investigation across diverse ethnic groups. Therefore, conclusions regarding the comparative efficacy of S1-containing treatment strategies should be interpreted with caution, as they may be most applicable to Asian patients.

In our study, neoadjuvant regimens incorporating ICIs have been demonstrating significant improvements in pCR [12–14, 16, 45, 46, 57]. Evidence suggests that GC/GEJC patients who receive neoadjuvant therapy and have a higher pCR have also improved OS [58–61]. To date, the KEYNOTE-585 trial remains the only study which reports final OS outcomes in this setting. Although the addition of pembrolizumab to chemotherapy extended the median OS from 55.7 to 71.8 months, the difference did not achieve statistical significance (HR: 0.86; 95% CI: 0.71–1.06) [62]. It is noteworthy that the chemotherapy backbone employed in this trial consisted of a fluoropyrimidine plus cisplatin doublet. Whether alternative chemotherapy regimens may further potentiate survival benefits warrants continued investigation. In terms of safety, the incidence of SAEs was comparable across the groups, ranging from 24.2% to 35.2%. SOX_Toripalimab, SAP_Apatinib_Camrelizumab, and XELOX were identified as regimens most frequently linked to SAEs. Hematologic toxicities were the most common SAEs across all three regimens, with thrombocytopenia predominating in the SOX_Toripalimab group, whereas leukopenia was the most frequent in both the SAP_Apatinib_Camrelizumab and XELOX groups. Additional research is warranted to comprehensively assess the safety of the foregoing regimens.

To our knowledge, this is the first NMA assessing OS outcomes of neoadjuvant immunotherapy in resectable GC/GEJC. Furthermore, a comparative and ranking analysis of the outcomes was conducted, exclusively including randomized controlled trials (RCTs). This study provides valuable evidence to guide the selection of optimal neoadjuvant strategies for patients with resectable GC/GEJC. Nevertheless, several limitations must be noted. First, some clinical trials have yet to reach their endpoints, resulting in incomplete data availability. Second, although most studies consistently defined pCR as ypT0 ypN0, minor heterogeneity existed in pathological assessment protocols and grading systems. Such variability should be taken into account when interpreting the comparative outcomes of the pooled analysis. Third, the included trials enrolled patients with both gastric and gastroesophageal junction cancers; some incorporated radiotherapy, whereas others did not. In addition, the network integrated both Phase II and Phase III studies. These sources of clinical and methodological heterogeneity may have influenced the pooled effect estimates and should be carefully considered when interpreting the findings.

## Conclusion

This study assesses and ranks neoadjuvant treatment regimens for locally advanced GC and GEJC through a BNMA. Based on current evidence, the DOX regimen appears to be the most promising for improving OS, the FLOT_Durvalumab combination holds the greatest potential for higher pCR rates, whereas the DOS_Apatinib combination shows the greatest potential for achieving higher R0 resection rates. Nonetheless, it is important to emphasize that the OS analysis was derived from a single study. Therefore, validation of these OS outcomes in large-scale, randomized phase III trials is essential before these findings can be routinely applied in clinical practice.

## Supplementary Information

Supplementary Material 1

Supplementary Material 2

Supplementary Material 3

Supplementary Material 4

## Data Availability

All data generated or analysed during this study are included in this published article and its supplementary information files.
