# Supplementary material for: Optimal neoadjuvant regimens for locally advanced gastric and gastroesophageal junction cancer: a systematic review and bayesian network meta-analysis
Source: World J Surg Oncol. 2025 Dec 24;24:57. doi: 10.1186/s12957-025-04151-z (PMC12849486; doi:10.1186/s12957-025-04151-z)
Supplement: Supplementary file 1 — Supplementary Material 1 [file 12957_2025_4151_MOESM1_ESM.docx]

**Supplementary material 1 Search strategy**

**Pubmed**

1 Neoadjuvant Therapy[MeSH Terms]

2 Preoperative Period[MeSH Terms]

3 Perioperative Period[MeSH Terms]

4 neoadjuvant chemotherapy[MeSH Terms]

5 neoadjuvant chemoradiotherapy[MeSH Terms]

6 neoadjuvant immunotherapy[MeSH Terms]

7 "neo adjuvant therapy"[Title/Abstract] OR "neo adjuvant treatment"[Title/Abstract] OR "Neoadjuvant Chemoradiation*"[Title/Abstract] OR "Neoadjuvant Chemoradiotherap*"[Title/Abstract] OR "Neoadjuvant Chemotherap*"[Title/Abstract] OR "Neoadjuvant Radiation*"[Title/Abstract] OR "Neoadjuvant Radiotherap*"[Title/Abstract] OR "Neoadjuvant Systemic Therap*"[Title/Abstract] OR "Neoadjuvant Systemic Treatment*"[Title/Abstract] OR "Neoadjuvant Therap*"[Title/Abstract] OR "neoadjuvant treatment*"[Title/Abstract] OR "neo-adjuvant chemotherapy"[Title/Abstract] OR "neoadjuvant chemotherapy"[Title/Abstract] OR "neo-adjuvant chemo-radiation therapy"[Title/Abstract] OR "neo-adjuvant chemo-radiotherapy"[Title/Abstract] OR "neo-adjuvant chemoradiation therapy"[Title/Abstract] OR "neo-adjuvant chemoradiotherapy"[Title/Abstract] OR "neoadjuvant chemo-radiation therapy"[Title/Abstract] OR "neoadjuvant chemo-radiotherapy"[Title/Abstract] OR "neoadjuvant chemoradiation therapy"[Title/Abstract] OR "neoadjuvant chemoradiotherapy"[Title/Abstract]

8 #1 OR #2 OR #3 OR #4 OR #5 OR #6 OR #7

9 Stomach Neoplasms[MeSH Terms]

10 Esophagogastric Junction[MeSH Terms]

11 stomach cancer[MeSH Terms]

12 gastroesophageal junction cancer[MeSH Terms]

13 "Cancer of Stomach"[Title/Abstract] OR "cancer of the cardia"[Title/Abstract] OR "cancer of the gastric antrum"[Title/Abstract] OR "cancer of the gastric body"[Title/Abstract] OR "cancer of the gastric fundus"[Title/Abstract] OR "Cancer of the Stomach"[Title/Abstract] OR "cardia cancer"[Title/Abstract] OR "Esophagogastric Junction"[Title/Abstract] OR "gastric cancer*"[Title/Abstract] OR "gastric malignanc*"[Title/Abstract] OR "Gastric Neoplasm*"[Title/Abstract] OR "Gastroesophageal Junction*"[Title/Abstract] OR "malignanc* of the stomach"[Title/Abstract] OR "malignant gastric neoplasm"[Title/Abstract] OR "malignant gastric tumor"[Title/Abstract] OR "malignant neoplasm* of the stomach"[Title/Abstract] OR "malignant tumo*r* of the stomach"[Title/Abstract] OR "pyloric cancer"[Title/Abstract] OR "stomach cancer*"[Title/Abstract] OR "stomach malignanc*"[Title/Abstract] OR "Stomach Neoplasm*"[Title/Abstract]

14 #9 OR #10 OR #11 OR #12 OR #13

15 #8 AND #14

16 #15 AND (random* or control*)

**Embase**

1 'neoadjuvant therapy'/exp

2 'preoperative period'/exp

3 'perioperative period'/exp

4 'neoadjuvant chemotherapy'/exp

5 'neoadjuvant chemoradiotherapy'/exp

6 'neoadjuvant chemoimmunotherapy'/exp

7 'neoadjuvant immunotherapy'/exp

8 'neoadjuvant immunochemotherapy'/exp

9 'neo adjuvant therapy':ab,ti,kw OR 'neo adjuvant treatment':ab,ti,kw OR 'neoadjuvant chemoradiation*':ab,ti,kw OR 'neoadjuvant chemoradiotherap*':ab,ti,kw OR 'neoadjuvant chemotherap*':ab,ti,kw OR 'neoadjuvant radiation*':ab,ti,kw OR 'neoadjuvant radiotherap*':ab,ti,kw OR 'neoadjuvant systemic therap*':ab,ti,kw OR 'neoadjuvant systemic treatment*':ab,ti,kw OR 'neoadjuvant therap*':ab,ti,kw OR 'neoadjuvant treatment*':ab,ti,kw OR 'neo-adjuvant chemotherapy':ab,ti,kw OR 'neoadjuvant chemotherapy':ab,ti,kw OR 'neo-adjuvant chemo-radiation therapy':ab,ti,kw OR 'neo-adjuvant chemo-radiotherapy':ab,ti,kw OR 'neo-adjuvant chemoradiation therapy':ab,ti,kw OR 'neo-adjuvant chemoradiotherapy':ab,ti,kw OR 'neoadjuvant chemo-radiation therapy':ab,ti,kw OR 'neoadjuvant chemo-radiotherapy':ab,ti,kw OR 'neoadjuvant chemoradiation therapy':ab,ti,kw OR 'neoadjuvant chemoradiotherapy':ab,ti,kw

10 #1 OR #2 OR #3 OR #4 OR #5 OR #6 OR #7 OR #8 OR #9

11 'stomach neoplasms'/exp

12 'esophagogastric junction'/exp

13 'stomach cancer'/exp

14 'gastroesophageal junction cancer'/exp

15 'gastroesophageal junction cancer'/exp

16 'cancer of stomach':ab,ti,kw OR 'cancer of the cardia':ab,ti,kw OR 'cancer of the gastric antrum':ab,ti,kw OR 'cancer of the gastric body':ab,ti,kw OR 'cancer of the gastric fundus':ab,ti,kw OR 'cancer of the stomach':ab,ti,kw OR 'cardia cancer':ab,ti,kw OR 'esophagogastric junction':ab,ti,kw OR 'gastric cancer*':ab,ti,kw OR 'gastric malignanc*':ab,ti,kw OR 'gastric neoplasm*':ab,ti,kw OR 'gastroesophageal junction*':ab,ti,kw OR 'malignanc* of the stomach':ab,ti,kw OR 'malignant gastric neoplasm':ab,ti,kw OR 'malignant gastric tumor':ab,ti,kw OR 'malignant neoplasm* of the stomach':ab,ti,kw OR 'malignant tumo*r* of the stomach':ab,ti,kw OR 'pyloric cancer':ab,ti,kw OR 'stomach cancer*':ab,ti,kw OR 'stomach malignanc*':ab,ti,kw OR 'stomach neoplasm*':ab,ti,kw

17 #11 OR #12 OR #13 OR #14 OR #15 OR #16

18 #10 AND #17

19 'random*':ab,ti,kw OR 'control*':ab,ti,kw

20 #18 AND #19

21 #20 AND 'Article'/it

**Cochrane**

#1 MeSH descriptor: [Neoadjuvant Therapy] explode all trees

#2 MeSH descriptor: [Preoperative Period] explode all trees

#3 MeSH descriptor: [Perioperative Period] explode all trees

#4 MeSH descriptor: [Neoadjuvant Therapy] explode all trees

#5 MeSH descriptor: [Neoadjuvant Therapy] explode all trees

#6 (‘neo adjuvant therapy' OR 'neo adjuvant treatment' OR 'Neoadjuvant Chemoradiation*' OR 'Neoadjuvant Chemoradiotherap*' OR 'Neoadjuvant Chemotherap*' OR 'Neoadjuvant Radiation*' OR 'Neoadjuvant Radiotherap*' OR 'Neoadjuvant Systemic Therap*' OR 'Neoadjuvant Systemic Treatment*' OR 'Neoadjuvant Therap*' OR 'neoadjuvant treatment*' OR 'neo-adjuvant chemotherapy' OR 'neoadjuvant chemotherapy' OR 'neo-adjuvant chemo-radiation therapy' OR 'neo-adjuvant chemo-radiotherapy' OR 'neo-adjuvant chemoradiation therapy' OR 'neo-adjuvant chemoradiotherapy' OR 'neoadjuvant chemo-radiation therapy' OR 'neoadjuvant chemo-radiotherapy' OR 'neoadjuvant chemoradiation therapy' OR 'neoadjuvant chemoradiotherapy’):ab,ti,kw

#7 #1 or #2 or #3 or #4 or #5 or #6

#8 MeSH descriptor: [Stomach Neoplasms] explode all trees

#9 MeSH descriptor: [Esophagogastric Junction] explode all trees

#10 MeSH descriptor: [Stomach Neoplasms] explode all trees

#11 (‘Cancer of Stomach' OR 'cancer of the cardia' OR 'cancer of the gastric antrum' OR 'cancer of the gastric body' OR 'cancer of the gastric fundus' OR 'Cancer of the Stomach' OR 'cardia cancer' OR 'Esophagogastric Junction' OR 'gastric cancer*' OR 'gastric malignanc*' OR 'Gastric Neoplasm*' OR 'Gastroesophageal Junction*' OR 'malignanc* of the stomach' OR 'malignant gastric neoplasm' OR 'malignant gastric tumor' OR 'malignant neoplasm* of the stomach' OR 'malignant tumo*r* of the stomach' OR 'pyloric cancer' OR 'stomach cancer*' OR 'stomach malignanc*' OR 'Stomach Neoplasm*’):ab,ti,kw

#12 #8 or #9 or #10 or #11

#13 #7 and #12

#14 ('random*' OR 'control’):ab,ti,kw

#15 #13 and #14

**WOS**

1 "TS=((Cancer of Stomach) OR (cancer of the cardia) OR (cancer of the gastric antrum) OR (cancer of the gastric body) OR (cancer of the gastric fundus) OR (Cancer of the Stomach) OR (cardia cancer) OR (Esophagogastric Junction) OR (gastric cancer*) OR (gastric malignanc*) OR (Gastric Neoplasm*) OR (Gastroesophageal Junction*) OR (malignanc* of the stomach) OR (malignant gastric neoplasm) OR (malignant gastric tumor) OR (malignant neoplasm* of the stomach) OR (malignant tumo*r* of the stomach) OR (pyloric cancer) OR (stomach cancer*) OR (stomach malignanc*) OR (Stomach Neoplasm*)) "

2 "TS=((neo adjuvant therapy) OR (neo adjuvant treatment) OR (Neoadjuvant Chemoradiation*) OR (Neoadjuvant Chemoradiotherap*) OR (Neoadjuvant Chemotherap*) OR (Neoadjuvant Radiation*) OR (Neoadjuvant Radiotherap*) OR (Neoadjuvant Systemic Therap*) OR (Neoadjuvant Systemic Treatment*) OR (Neoadjuvant Therap*) OR (neoadjuvant treatment*) OR (neo-adjuvant chemotherapy) OR (neoadjuvant chemotherapy) OR (neo-adjuvant chemo-radiation therapy) OR (neo-adjuvant chemo-radiotherapy) OR (neo-adjuvant chemoradiation therapy) OR (neo-adjuvant chemoradiotherapy) OR (neoadjuvant chemo-radiation therapy) OR (neoadjuvant chemo-radiotherapy) OR (neoadjuvant chemoradiation therapy) OR (neoadjuvant chemoradiotherapy)) "

3 "TS=((random*) or (control*))"

4 "#1 and #2"

5 "#3 and #4"
