# Supplementary material for: Optimal neoadjuvant regimens for locally advanced gastric and gastroesophageal junction cancer: a systematic review and bayesian network meta-analysis
Source: World J Surg Oncol. 2025 Dec 24;24:57. doi: 10.1186/s12957-025-04151-z (PMC12849486; doi:10.1186/s12957-025-04151-z)

**Supplementary Figures**

Figure S1. OS Node Thunderbolt


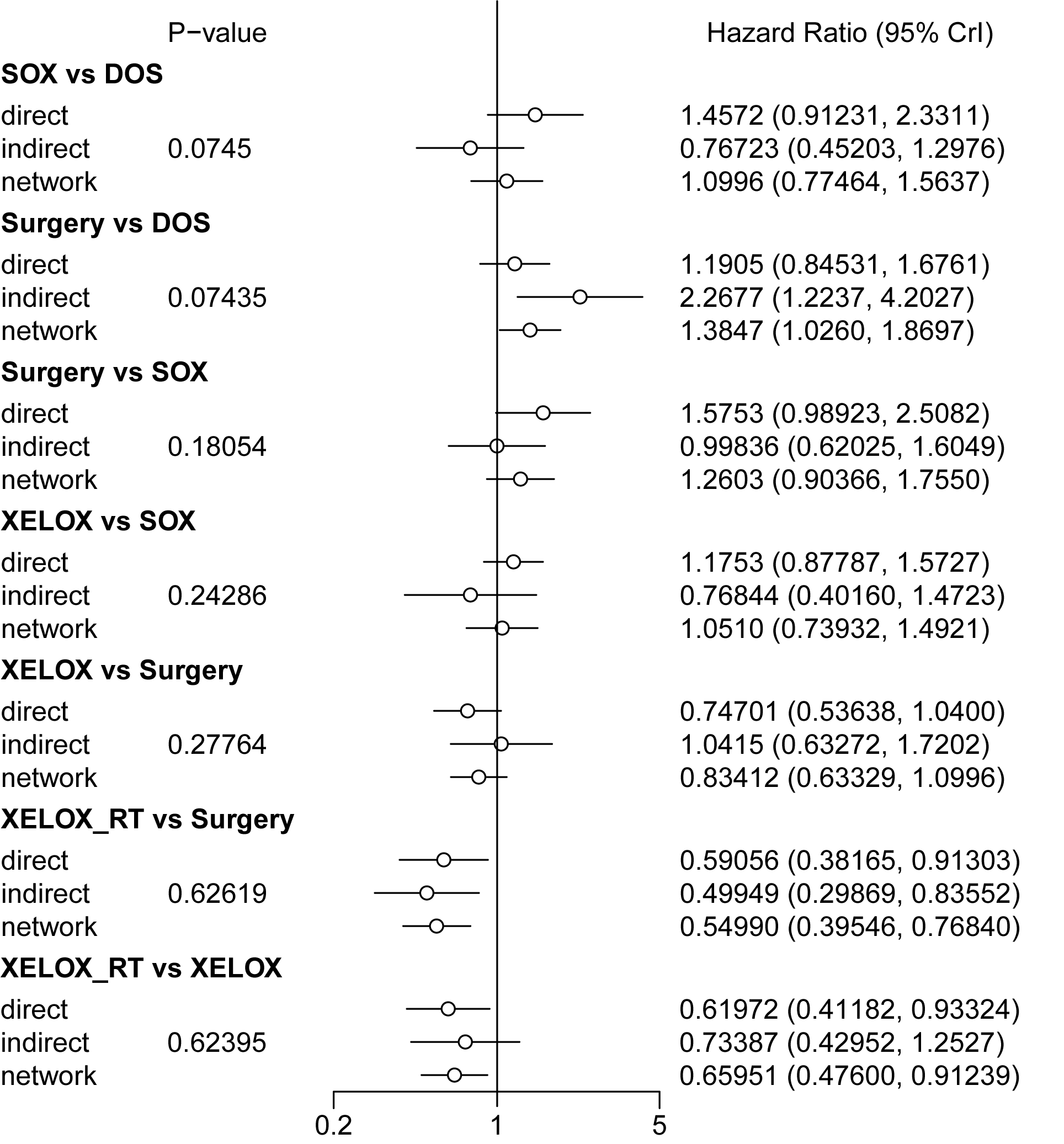


Figure S2. Funnel plot of OS, pCR, R0. (a) OS funnel graph, (b) pCR Funnel Diagram, (c) R0 Funnel Diagram


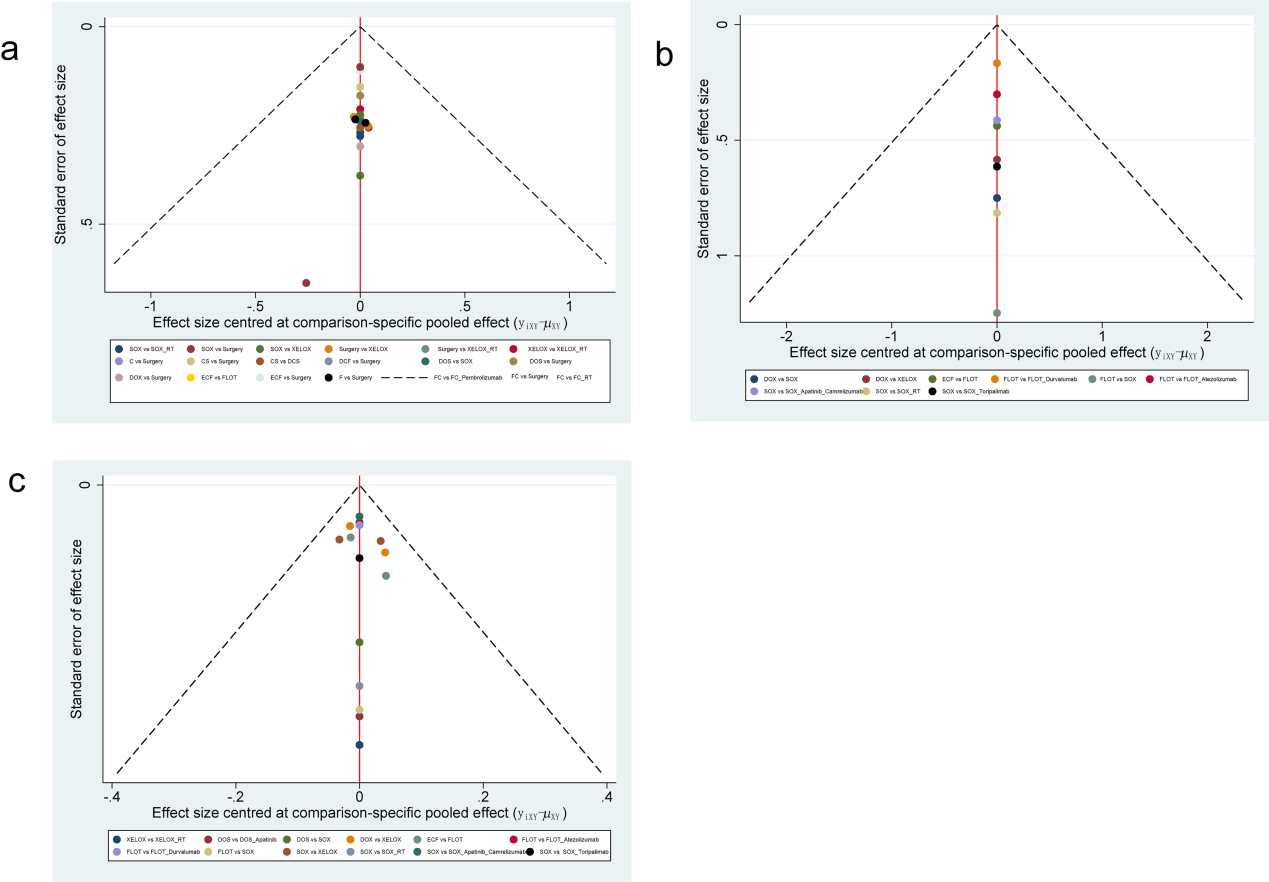


Figure S3. Quality Assessment


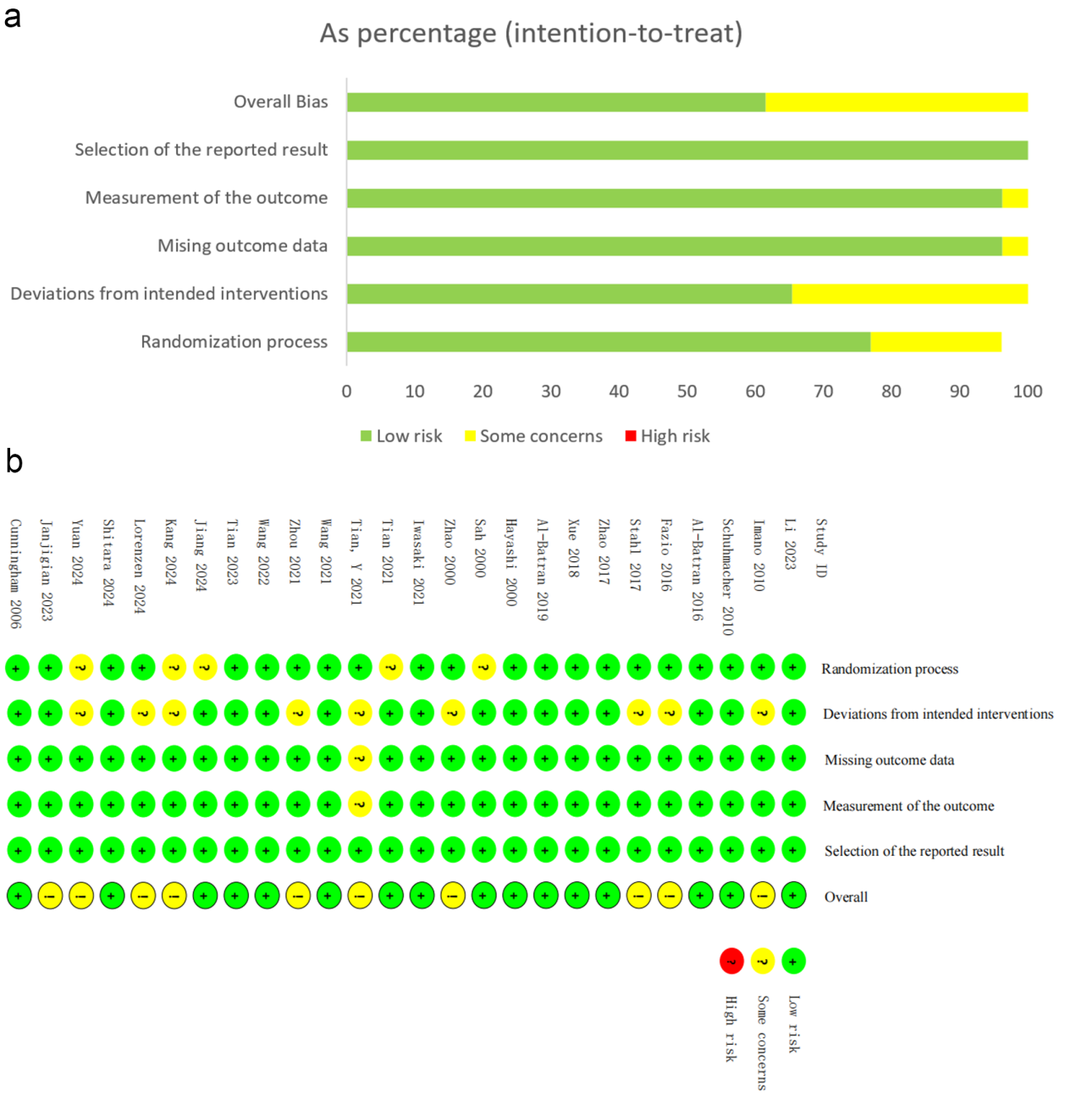

Supplement: Supplementary file 3 — Supplementary Material 3 [file 12957_2025_4151_MOESM3_ESM.docx]
