# Supplementary material for: Optimal neoadjuvant regimens for locally advanced gastric and gastroesophageal junction cancer: a systematic review and bayesian network meta-analysis
Source: World J Surg Oncol. 2025 Dec 24;24:57. doi: 10.1186/s12957-025-04151-z (PMC12849486; doi:10.1186/s12957-025-04151-z)
Supplement: Supplementary file 4 — Supplementary Material 4 [file 12957_2025_4151_MOESM4_ESM.docx]

**Table S1 Dails of the pharmacological treatment protocol of included trials**

| Name of Protocol | Details of Protocol |
| --- | --- |
| C | Cisplatin |
| CS | Cisplatin;S-1 |
| DCF | Docetaxel;Cisplatin;Fluorouracil |
| DCS | Docetaxel;Cisplatin;S-1 |
| DOS | Docetaxel;Oxaliplatin;S-1 |
| DOS_Apatinib | Docetaxel;Oxaliplatin;S-1;Apatinib |
| DOX | Docetaxel;Oxaliplatin;Capecitabine |
| ECF | Epirubicin;Cisplatin;Fluorouracil |
| F | Fluorouracil |
| FC | Fluorouracil;Cisplatin |
| FC_RT | Fluorouracil;Cisplatin;Radiotherapy |
| FLOT | Fluorouracil;Leucovorin;Oxaliplatin;Docetaxel |
| FLOT_Atezolizumab | Fluorouracil;Leucovorin;Oxaliplatin;Docetaxel;Atezolizumab |
| FLOT_Durvalumab | Fluorouracil;Leucovorin;Oxaliplatin;Docetaxel;Durvalumab |
| FC_Pembrolizumab | Fluorouracil;Cisplatin;Pembrolizumab |
| SAP | S-1;Nab-paclitaxel |
| SAP_Apatinib_Camrelizumab | S-1;Nab-paclitaxel;Apatinib;Camrelizumab |
| SOX | S-1;Oxaliplatin |
| SOX_Apatinib_Camrelizumab | S-1;Oxaliplatin;Apatinib;Camrelizumab |
| SOX_RT | S-1;Oxaliplatin;Radiotherapy |
| SOX_Toripalimab | S-1;Oxaliplatin;Toripalimab |
| XELOX | Capecitabine;Oxaliplatin |
| XELOX_RT | Capecitabine;Oxaliplatin;Radiotherapy |

**Table S2 Grade ≥3 Treatment-related adverse events (TRAEs)**

| Grade≥3 Treatment-related adverse events(TRAEs) | | | |
| --- | --- | --- | --- |
| Study | Treatment | Responders No(%) | Main TRAEs type No(%) |
| Shitara 2025 | FC_Pembrolizumab | 102(25.6%) | Neutrophil count decreased 103(26%), Neutropenia 70(17%), Nausea 24 (6%) |
|  | FC | 97(24.2%) | Neutrophil count decreased 92(24%), Neutropenia 68(17), Anaemia 30(7%) |
| Yuan 2024 | SOX_Toripalimab | 19(35.2%) | Thrombocytopenia 10(18.5%), Neutropenia 9(16.7%), Leukopenia 4(7.4%) |
|  | SOX | 16(29.6%) | Thrombocytopenia 7(13%), Neutropenia 7(13%), Leukopenia 6(11.1%) |
| Al-Batran 2019 | ECF | 96(26.9%) | Neutropenia 139 (39%), Leukopenia 75 (21%), Alopecia 74 (21%) |
|  | FLOT | 97(27.2%) | Neutropenia 181 (51%), Alopecia 98 (28%), Leukopenia 94 (27%) |
| Tian 2021 | DOX | 38(32.2%) | Neutropenia 30(32.3%), Leucopenia 20(21.5%), Nausea 14(15.1%) |
|  | XELOX | 30(32.6%) | Neutropenia 28(30.4%), Leucopenia 21(22.8%), Nausea 15(16.3%) |
| Lin 2024 | SAP_Apatinib_Camrelizumab | 17(33.3%) | Leukopenia 7(13.7%), Neutropenia 5(9.8%), ALT elevation 5(9.8%) |
|  | SAP | 14(26.4%) | Leukopenia 4(7.5%), Neutropenia 3 (5.7%), ALT elevation 3 (5.7%) |
